# Interaction of Virus in Cancer Patients: A Theoretical Dynamic Model

**DOI:** 10.3390/bioengineering10020224

**Published:** 2023-02-07

**Authors:** Veli B. Shakhmurov, Muhammet Kurulay, Aida Sahmurova, Mustafa Can Gursesli, Antonio Lanata

**Affiliations:** 1 Department of Industrial Engineering, Antalya Bilim University, Ciplakli Mahallesi Farabi Caddesi 23 Dosemealti, Antalya 07190, Turkey; 2 Center of Analytical-Information Resource, Azerbaijan State Economic University, 194 M. Mukhtarov, Baku AZ1001, Azerbaijan; 3 Department of Mathematics Engineering, Yildiz Technical University, Istanbul 34225, Turkey; 4 Department of Nursing, Antalya Bilim University, Ciplakli Mahallesi Farabi Caddesi 23 Dosemealti, Antalya 07190, Turkey; 5 Department of Information Engineering, University of Florence, Via Santa Marta 3, 50139 Firenze, Italy; 6 Department of Education, Literatures, Intercultural Studies, Languages and Psychology, University of Florence, 50135 Florence, Italy

**Keywords:** mathematical modeling, virus, immune system cells, tumor growth, stability of dynamical systems

## Abstract

This study reports on a phase-space analysis of a mathematical model of tumor growth with the interaction between virus and immune response. In this study, a mathematical determination was attempted to demonstrate the relationship between uninfected cells, infected cells, effector immune cells, and free viruses using a dynamic model. We revealed the stability analysis of the system and the Lyapunov stability of the equilibrium points. Moreover, all endemic equilibrium point models are derived. We investigated the stability behavior and the range of attraction sets of the nonlinear systems concerning our model. Furthermore, a global stability analysis is proved either in the construction of a Lyapunov function showing the validity of the concerned disease-free equilibria or in endemic equilibria discussed by the model. Finally, a simulated solution is achieved and the relationship between cancer cells and other cells is drawn.

## 1. Introduction

The nonlinearity approach has been shown to be powerful in revealing unexpected dynamics in cancer growth processes, manifested by different responses of the dynamics to different concentrations of immune cells at different stages of cancer growth development [1,2,3,4,5,6,7,8,9,10,11,12]. Research findings have highlighted the complex nature of the processes and their interaction behind the cancer growth [13]. Taking into account all these complex processes behind cancer growth, the introduction of nonlinear mathematical models can balance and minimize the inconsistencies among the different already proposed mathematical models that are related to the influence of anticancer factors on cancer growth. The computation of mathematical non-spatial models of cancer tumor growth in the broad context of studies of tumor-immune interactions is one of the intensively developing areas in modern mathematical biology [1,2,3,4,5,6,7,8,9].

Currently, one of the most challenging research issue is represented by the formalization of the interactions among uninfected cells, free viruses, and immune responses. In this context, the dynamic models could still play a crucial role [14,15,16,17]. One of these models, a three-dimensional dynamic model of viral infection, was proposed by Nowak et al. [15,16,17]. The aforementioned model is capable of generalizing numerical methods of autonomous dynamical systems. Moreover, Giesl [18] characterized a Lyapunov function as a solution for a suitable linear first-order partial differential equation and approximated it by using radial basis functions.

Furthermore, Yang and Wang [19] proposed a mathematical model which, employing non-constant transmission rates, is able to take into account both the environmental and epidemiological conditions, reflecting the impact of endemic disease. They have acknowledged the challenge of designing mathematical models of virus dynamics description. As a matter of fact, several models have been produced, leading sometimes to different estimates. They have devised a deterministic compartmental (SEIR) model. Moreover, endemic outbreaks (e.g., COVID-pandemic [20,21]) will continue to grow and peak in time, due to practically implemented public health interventions. Moreover, recent discoveries showed that the best solution is predominantly permanent and rigid self-isolation. However, the necessity of new interventions cannot be neglected. In this framework, we propose a deterministic compartmental model based on SEIR model [22] to describe the dynamics of the virus contribution to the spectrum of tumor-immune interaction.

Tang et al. provided a detailed analysis of the SEIR model and showed its applications by using publicly disclosed data. Among other findings, analytical and numerical results indicate that virus infection will remain endemic and require long-term disease prevention and intervention programs. Then, a new spatial approach (SBDiEM) for infectious dynamic prediction, and mathematical epidemiology models have been shown helpful in contrasting epidemic outbreaks [23]. Moreover, the model can be adjusted to identify past outbreaks and viruses. Methodologies can have important implications for national health systems, international stakeholders, and policymakers with the aim of developing epidemic control, vaccination, and prevention strategies. The model can be embedded in a global AI surveillance system to contrast outbreaks. Bekirosa et al. [22] investigated the transmission dynamics of viruses and a separated mathematical model between humans in different regions. It showed that protecting vulnerable individuals, preventing contact with infected people, and controlling incentives to join quarantine centers provide the most cost-effective strategy to control the disease. In addition, the most appropriate campaigns should be carried out by preventing people from moving from one region to another, encouraging them to attend quarantine centers, conducting awareness campaigns aimed at being affected by viruses, safety campaigns and health measures. Khajji et al. presented the implementation of a global network model with the local epidemic SEIR model to measure the epidemic dynamics of COVID-19 in China and the USA [24]. Researchers demonstrated how mathematical modeling can help in estimating the outbreak dynamics and provide decision guidelines for successful outbreak control. The model can become a valuable tool for evaluating the potential of vaccination and quantifying the effect of relaxing political measures including total lockdown, shelter-in-place, and travel restrictions for low-risk subgroups of the population or for the population as a whole [25]. It is worthwhile noting that the mathematical models identified by the World Health Organization (WHO) can play an important role in providing evidence-based information to healthcare decision-makers and policymakers. Moreover, the modeling approach can assist in understanding the spread of viruses in the population. As a matter of fact, research findings also evidenced that several viruses are linked with cancer in humans [26]. In this work, we have created a mathematical model of virus transmission based on the SEIR model. Furthermore, our study includes mathematical models of the relationship between cancer cells and viruses. In the context of the therapy, some numerical cases, by Pham et al., demonstrated that a dynamic, time-delayed SEIR model can be used to monitor the effects of chemotherapy drug therapy and the growth rate of tumor virus-infected cells and autoimmune disease [27]. The results of modeling suggest determining the progression of tumor cells in the human body based on partial differential equations under the influence of chemotherapy, autoimmune diseases and time delays. Hence, the model can also be used to predict when the free state of tumor viruses will be reached as time progresses, and to predict the state of healthy cells in the body as time progresses. In addition, Gao et al. proved the existence and uniqueness of the solution, the system stability, along with the local stability and global stability of infection-free homeostasis. Moreover, they also examined the uniform persistence and local stability of the infected state and demonstrated, through the Creation of the Lyapunov function, the global stability of the infected state. Finally, the theoretical results were verified by numerical simulation [28]. Qian Lia et al. showed a new mathematical modeling framework based on the latency of differential equation to study tumor virotherapy with antitumor immunity mediated by oncolytic viruses involving complex tumor-virus-immune system interactions [29]. Baleanu et al., provided a generalized fractional model to analyze, control and synchronize the associated hyper-chaotic behaviors by means of a variety of approaches. More specifically, the relevant nonlinear mathematical model was presented in the form of both integer and fractional degree differential equations [30,31]. Yasmin implemented an epidemic model to conceptualize the phenomenon of the transmission of pneumococcal pneumonia by vaccination and treatment factors [32]. Given the literature of nonlinear dynamic systems, here, we propose a further mathematical model concerning to the initial value problem for the following nonlinear systems. Modeling can help better understand a virus spreading in the population. Our study also includes mathematical models of the relationship between cancer cells and viruses.

(1) $$\dot{I}\left(t\right)=\beta \frac{T\left(t\right)I\left(t\right)}{T\left(t\right)+k_{1}}+\beta_{1}I\left(t\right)-q_{13}E\left(t\right)I\left(t\right),$$

(2) $$\dot{T}=r_{2}T\left(1-k_{2}^{-1}T\right)+\beta_{2}V\left(t\right)T-q_{23}ET,$$

(3) $$\dot{E}\left(t\right)=\frac{d_{1}I\left(t\right)E\left(t\right)}{I\left(t\right)+k_{3}}-q_{33}I\left(t\right)E\left(t\right)-d_{2}E\left(t\right),$$

(4) $$\dot{V}\left(t\right)=dnE\left(t\right)-cV\left(t\right),\;$$

$$I\left(t_{0}\right)=I_{0},\;T\left(t_{0}\right)=T_{0},\;E\left(t_{0}\right)=E_{0},$$

$$V\left(t_{0}\right)=V_{0},\;t_{0}\in \left[0,\right.\left.t_{0}\right),$$

where $I=I\left(t\right)$, $T=T\left(t\right)$, $E=E\left(t\right)$ and $V=V\left(t\right)$ denote the concentration of infected cells, cancer cells, effector immune cells and free viruses at time $t\in \left[0,\right.\left.t_{0}\right)$, respectively. In the first equation, the interaction dynamic of infected and cancer cells are given by the rational function which depends on the virus concentration with positive constants β and $k_{1}$. They are respectively maximal I cells activation rate by contact with tumor cells T and half saturation constant. The constants here, $\beta_{1}>0$, $q_{13}>0$ are growth and decrease rates, rate of the infected cells due to viruses and death rate due to immune effect, respectively. The first term of the second equation corresponds to the logistic growth of tumor cells in the absence of any effect from other cells populations with the growth rate of $r_{2}$ and maximum carrying capacity $k_{2}$. Here, competition between tumor cells $T\left(t\right)$ with virus and effector immune cells which results in the growth and loss of the tumor cells population is given by terms $\beta_{2}V\left(t\right)T$, $q_{23}ET$; here $\beta_{2}$ (rate of T produced by V) and $q_{23}$ (killing rate of T cells by E cells) are positive numbers. Viruses can cause cancer by direct and indirect modes of action(see, e.g., [33]). They studied the local and global dynamics model of cancer tumor growth [34]. Next, the parameter $q_{33}$ refers to the killing rate of the infected cells rate by the immune cells $E\left(t\right)$. Moreover, the dynamic of effector immune cells (recognition process) is given by the rational function which depends on the virus concentration with positive constants $k_{3}$ and $d_{1}$. Where $k_{3}$ and $d_{1}$ are respectively half-saturation constant and maximal $E\left(t\right)$ cells activation rate by contact with $I\left(t\right)$ cells. The effector immune cells die naturally at the rate $d_{2}$. The infected cells produce new viruses, V(t), at the rate dn during their life, on average having the length $\frac{1}{d}$, where *n*>0 is some integer number. The constant c>0 is the rate at which the viruses are cleared, and the average lifetime of a free virus is $\frac{1}{c}$.

## 2. Boundedness and Dissipativity

In this section, we shall show that the model is bounded with negative divergence, positively invariant with respect to a region in $R_{+}^{4}$ and dissipative. As we are interested in biologically relevant solutions of the system, the next results show that the positive octant is invariant and that the upper limits of trajectories depend on the parameters.

We put

$$I\left(t\right)=x_{1}\left(t\right),\;T\left(t\right)=x_{2}\left(t\right),\;E\left(t\right)=x_{3}\left(t\right),\;V\left(t\right)=x_{4}\left(t\right).$$

Then the problem (1) and (2) is reduced the following form:

(5) $${\dot{x}}_{1}\left(t\right)=f_{1}\left(x\right),\;{\dot{x}}_{2}\left(t\right)=f_{2}\left(x\right),\;{\dot{x}}_{3}\left(t\right)=f_{3}\left(x\right),\;{\dot{x}}_{4}\left(t\right)=f_{4}\left(x\right),$$

(6) $$x_{1}\left(t_{0}\right)=x_{10},\;x_{2}\left(t_{0}\right)=x_{20},\;x_{3}\left(t_{0}\right)=x_{30},$$

$$x_{4}\left(t_{0}\right)=x_{40},\;t_{0}\in \left[0,\right.\left.T\right),$$

where,

(7) $$x=x\left(t\right)=\left(x_{1},x_{2},x_{3},x_{4}\right),\;x_{k}=x_{k}\left(t\right),\;k=1,2,3,4,$$

$$f_{1}\left(x\right)=\beta \frac{x_{2}\left(t\right)x_{1}\left(t\right)}{x_{2}\left(t\right)+k_{1}}+\beta_{1}\;x_{1}\left(t\right)-q_{13}x_{3}\left(t\right)x_{1}\left(t\right),$$

$$f_{2}\left(x\right)=r_{2}x_{2}\left(1-k_{2}^{-1}x_{2}\right)+\beta_{2}x_{4}\left(t\right)x_{2}-q_{23}x_{3}x_{2},$$

$$f_{3}\left(x\right)=\frac{d_{1}x_{1}\left(t\right)x_{3}\left(t\right)}{x_{1}\left(t\right)+k_{3}}-q_{33}x_{1}\left(t\right)x_{3}\left(t\right)-d_{2}x_{3}\left(t\right),\;$$

$$f_{4}\left(x\right)=dnx_{3}\left(t\right)-cx_{4}\left(t\right).$$

Let

$$R_{+}^{4}=\left\{x=\left(x_{1},x_{2},x_{3},x_{4}\right)\in R^{4},\;x_{k}>0\right\}.$$

**Condition 1.**  *Let $d_{1}\leq k_{3}q_{33}$, $d_{2}>r_{2}$ and $\beta \leq k_{1}k_{2}^{-1}$. Consider the problem (5)–(6) with $t_{0}=0.$*

**Theorem 1.**  *Assume that the Condition 1 holds. Then the system (6) is with the negative divergence and is dissipative.*

**Proof.**  Indeed, from $\left(6\right)$ we have

$$\frac{\partial f_{1}}{\partial x_{1}}+\frac{\partial f_{2}}{\partial x_{2}}+\frac{\partial f_{3}}{\partial x_{3}}+\frac{\partial f_{4}}{\partial x_{4}}=\frac{\beta x_{2}}{x_{2}+k_{1}}+\beta_{1}-q_{13}x_{3}+$$

$$r_{2}-2r_{2}\left(k_{2}^{-1}x_{2}\right)+\beta_{2}x_{4}-q_{23}x_{3}+\frac{d_{1}x_{1}}{x_{1}+k_{3}}-q_{33}x_{1}-d_{2}-c$$

$$=\left[\frac{d_{1}}{x_{1}+k_{3}}-q_{33}\right]x_{1}+\left[\frac{\beta }{x_{2}+k_{1}}-2r_{2}k_{2}^{-1}\right]x_{2}$$

$$-\left(q_{13}+q_{23}\right)x_{3}+\beta_{2}x_{4}+r_{2}+\beta_{1}-c-d_{2}.$$

Hence, by Condition 1 the system $\left(5\right)$ is dissipative on the domain

$$\Omega =\left\{x\in R_{+}^{4}:\right.\;\left(q_{13}+q_{23}\right)x_{3}+\left(\beta_{1}+\beta_{2}\right)x_{4}\leq \left.d_{2}-r_{2}\right\}.$$

□

## 3. The Local Stability of Equilibria Points

In this section, we will derive the stability properties of equilibria points of the system $\left(5\right)$. Let

$$\;B_{r}\left(\bar{x}\right)=\left\{x\in R^{4},\;{∥x-\bar{x}∥}_{R^{3}}<r\right\}.$$

**Condition 2.**  *Let the following assumptions hold:*

(8) $${\left(k_{3}q_{33}+d_{2}-d_{1}\right)}^{2}\geq 4q_{33}k_{3}d_{2}.$$

**Theorem 2.**  *Assume that the Condition 2 is satisfied. The points $P_{0}=P_{0}\left(0,0,0,0\right)$, $P_{i}=P_{i}\left(x_{1i},0,\frac{\beta_{1}}{q_{13}},\frac{\beta_{1}dn}{cq_{13}}\right)$, i=1,2 and $P_{3}=P_{3}\left(0,k_{2}r_{2},0,0\right)$ are the equilibria points of the system $\left(5\right)$ in $R_{+}^{4}$.*

**Proof.**  In view of $\left(5\right)$ and $\left(7\right)$, equilibria points of $\left(5\right)$ are the solutions of the following system

(9) $$\left[\frac{\beta x_{2}}{x_{2}+k_{1}}+\beta_{1}-q_{13}x_{3}\right]x_{1}=0,$$

$$\left[r_{2}\left(1-k_{2}^{-1}x_{2}\right)+\beta_{2}x_{4}-q_{23}x_{3}\right]x_{2}=0,$$

$$\left[\frac{d_{1}x_{1}}{x_{1}+k_{3}}-q_{33}x_{1}-d_{2}\right]x_{3}=0,\;dnx_{3}-cx_{4}=0.$$

From $\left(9\right)$ it is clear to see that the point $P_{0}=\left(0,0,0,0\right)$ is equilibria point of $\left(5\right)$. Moreover, the other solutions of $\left(9\right)$ can be derived from the following equations

$$\frac{\beta x_{2}}{x_{2}+k_{1}}+\beta_{1}-q_{13}x_{3}=0,\;r_{2}\left(1-k_{2}^{-1}x_{2}\right)+\beta_{2}x_{4}-q_{23}x_{3}=0,$$

(10) $$\frac{d_{1}x_{1}}{x_{1}+k_{3}}-q_{33}x_{1}-d_{2}=0\;,\;dnx_{3}-cx_{4}=0.$$

Let $x_{1}\neq 0$, $x_{2}=0$. From the first and forth equations of $\left(10\right)$, we get

(11) $$x_{3}=\frac{1}{q_{13}}\left[\frac{\beta x_{2}}{x_{2}+k_{1}}+\beta_{1}\right]=\frac{\beta_{1}}{q_{13}},\;x_{4}=\frac{\beta_{1}dn}{cq_{13}}.$$

Moreover from the third equation for $x_{3}\neq 0$ we have

(12) $$\;\nu_{1}x_{1}^{2}+\nu_{2}x_{1}+\nu_{3}=0,$$

where

$$\;\nu_{1}=q_{33},\;\nu_{2}=k_{3}q_{33}+d_{2}-d_{1},\;\nu_{3}=k_{3}d_{2}.$$

By Condition 2,

$$\nu_{2}^{2}-4\nu_{1}\nu_{3}\geq 0.$$

Thus by solving $\left(12\right)$, we have

(13) $$\;x_{11}=\frac{-\nu_{2}+\sqrt{\nu_{2}^{2}-4\nu_{1}\nu_{3}}}{2\nu_{1}},\;x_{12}=\frac{-\nu_{2}-\sqrt{\nu_{2}^{2}-4\nu_{1}\nu_{3}}}{2\nu_{1}}.$$

Let now $x_{1}=x_{3}=x_{4}=0$ and $x_{2}\neq 0$. Then from the second equation $\left(9\right)$, we obtain $x_{2}=k_{2}r_{2}$, i.e., we get that the point $E_{4}\left(0,k_{2}r_{2},0,0\right)$ is also a stable point for the system $\left(5\right)$.

Hence, from $\left(11\right)$, we obtain that the points $P_{i}\left(x_{1i},0,\frac{\beta_{1}}{q_{13}},\frac{\beta_{1}dn}{cq_{13}}\right)$, i=1,2 are stabile points for $\left(5\right).$ □

**Remark 1.**  *Note that, these points are biologically feasible equilibria, when all coordinates are nonnegative, i.e.,*

$$\frac{-\nu_{2}\pm \sqrt{\nu_{2}^{2}-4\nu_{1}\nu_{3}}}{2\nu_{1}}\geq 0.$$

Consider now, the linearized matrix of $\left(5\right)$, i.e., the Jacobian matrix according to system $\left(5\right)$:

$$\frac{Df}{Dx}=\left[\begin{array}{cccc} \frac{\partial f_{1}}{\partial x_{1}} & \frac{\partial f_{1}}{\partial x_{2}}\; & \frac{\partial f_{1}}{\partial x_{3}} & \frac{\partial f_{1}}{\partial x_{4}}\\ \frac{\partial f_{2}}{\partial x_{1}} & \frac{\partial f_{2}}{\partial x_{2}}\; & \frac{\partial f_{2}}{\partial x_{3}} & \frac{\partial f_{2}}{\partial x_{4}}\\ \frac{\partial f_{3}}{\partial x_{1}} & \frac{\partial f_{3}}{\partial x_{2}}\; & \frac{\partial f_{3}}{\partial x_{3}} & \frac{\partial f_{3}}{\partial x_{4}}\\ \frac{\partial f_{4}}{\partial x_{1}} & \frac{\partial f_{4}}{\partial x_{2}}\; & \frac{\partial f_{4}}{\partial x_{3}} & \frac{\partial f_{4}}{\partial x_{4}} \end{array}\right]=\left[\begin{array}{cccc} d_{11}\left(x\right) & d_{12}\left(x\right) & d_{13}\left(x\right) & 0\\ 0 & d_{22}\left(x\right) & d_{23}\left(x\right) & 0\\ d_{31}\left(x\right) & 0 & d_{33}\left(x\right) & 0\\ 0 & dn & 0 & -c \end{array}\right],$$

where

$$d_{11}\left(x\right)=\frac{\beta x_{2}}{x_{2}+k_{1}}+\beta_{1}-q_{13}x_{3},\;d_{12}\left(x\right)=\frac{\beta kx_{1}}{{\left(x_{2}+k_{1}\right)}^{2}},$$

(14) $$\;d_{13}\left(x\right)=-\frac{\beta kq_{13}x_{1}}{{\left(x_{2}+k_{1}\right)}^{2}},\;d_{14}\left(x\right)=\frac{\beta \beta_{1}kx_{1}}{{\left(x_{2}+k_{1}\right)}^{2}},\;$$

$$d_{22}\left(x\right)=r_{2}\left(1-2k_{2}^{-1}x_{2}\right)+\beta_{2}x_{4}-q_{23}x_{3},\;d_{23}\left(x\right)=-q_{23}x_{2},$$

$$d_{31}\left(x\right)=\left[\frac{d_{1}k_{3}}{{\left(x_{1}+k_{3}\right)}^{2}}-q_{33}\right]x_{3},\;d_{33}\left(x\right)=\frac{d_{1}x_{1}}{x_{1}+k_{3}}-q_{33}x_{1}-d_{2}.$$

Then, the Jacobian matrix of $\left(5\right)$ at the point $P_{0}$ is

(15) $$A_{0}=\frac{Df}{Dx}\left(0\right)=\left[\begin{array}{cccc} 0 & 0 & 0 & 0\\ 0 & r_{2} & 0 & 0\\ 0 & 0 & -d_{3} & 0\\ 0 & dn & 0 & -c \end{array}\right].$$

Note that the linearized matrices of $\left(5\right)$ according to other stability points $P_{i}$ are the following:

(16) $$A_{i}=\left[\begin{array}{cccc} d_{11}\left(P_{i}\right) & d_{12}\left(P_{i}\right) & d_{13}\left(P_{i}\right) & 0\\ 0 & d_{22}\left(P_{i}\right) & d_{23}\left(P_{i}\right) & 0\\ d_{31}\left(P_{i}\right) & 0 & d_{33}\left(P_{i}\right) & 0\\ 0 & dn & 0 & -c \end{array}\right],\;i=1,2.$$

The linearized matrices of $\left(5\right)$ according to other stability points $P_{3}\left(0,k_{2}r_{2},0,0\right)$ is the following

(17) $$A_{3}=\left[\begin{array}{cccc} d_{11}\left(P_{3}\right) & d_{12}\left(P_{3}\right) & d_{13}\left(P_{3}\right) & 0\\ 0 & d_{22}\left(P_{3}\right) & d_{23}\left(P_{3}\right) & 0\\ d_{31}\left(P_{3}\right) & 0 & d_{33}\left(P_{3}\right) & 0\\ 0 & dn & 0 & -c \end{array}\right],$$

where

$$d_{11}=\frac{\beta k_{2}r_{2}}{k_{2}r_{2}+k_{1}}+\beta_{1},\;d_{12}=0,$$

(18) $$\;d_{13}=0,\;d_{14}=0,\;d_{22}=r_{2}\left(1-2k_{2}^{-1}k_{2}r_{2}\right),$$

$$\;d_{23}=-q_{23}k_{2}r_{2},\;d_{31}=0,\;d_{33}=-d_{2}.$$

**Condition 3.**  *Assume the following assumptions are satisfied*

$$d_{22}<0,\;\left(d_{11}+d_{33}\right)\leq 0,\;{\left(d_{11}-d_{33}\right)}^{2}\geq 4d_{31}d_{12}^{2},\;d_{3}\geq k_{3}q_{32},$$

$$\left(d_{11}+d_{33}\right)\pm \sqrt{{\left(d_{11}-d_{33}\right)}^{2}-4d_{31}d_{12}^{2}}\leq 0.$$

*Let $d_{jk}=d_{jk}\left(P_{i}\right)$ for i=1,2. We show here, the following results.*

**Theorem 3.**  *The point $E_{0}$ is a saddle point for the system of $\left(5\right).$*

**Proof.**  Indeed, it is clear that $\lambda_{1}=0$, $\lambda_{2}=r_{2}$, $\lambda_{3}=-d_{3}$ and $\lambda_{4}=-c$ are the eigenvalues of the matrix $A_{0}$. Since $r_{2}$, $d_{3}$, *c* are positive, all eigenvalues of $A_{0}$ are non positive, i.e., $A_{0}$ is a saddle point for the linearized system of $\left(5\right).$ □

**Theorem 4.**  *Let Conditions 2 and 3 hold. Then $P_{i}$ are the locally stable points for the system of $\left(5\right)$. Moreover, $P_{i}$ are saddle points, when $d_{22}\geq 0$, $\left(d_{11}+d_{33}\right)\geq 0$ and $d_{11}d_{33}+d_{31}d_{12}^{2}\geq 0.$*

**Proof.**  The eigenvalues of the matrices $A_{i}$ can found as the solutions of the following equations

$$A_{i}-\lambda I=\left[\begin{array}{cccc} d_{11}-\lambda & d_{12} & d_{13} & 0\\ 0 & d_{22}-\lambda & d_{23} & 0\\ d_{31} & 0 & d_{33}-\lambda & 0\\ 0 & dn & 0 & -c-\lambda \end{array}\right]$$

(19) $$=\left(c+\lambda \right)\left[\begin{array}{ccc} d_{11}-\lambda & d_{12} & d_{13}\\ 0 & d_{22}-\lambda & d_{12}\\ d_{31} & 0 & d_{33}-\lambda \end{array}\right]$$

$$=\;\left(c+\lambda \right)\left[\prod_{k=1}^{3}\left[\left(d_{kk}-\lambda \right)\right]+d_{31}d_{12}^{2}-d_{13}d_{31}\left(d_{22}-\lambda \right)\right]=0.$$

Hence $\lambda_{1}=-c$ is a eigenvalue of $A_{i}$, and other eigenvalues are as the solution of the equation

(20) $$\prod_{k=1}^{3}\left[\left(d_{kk}-\lambda \right)\right]+d_{31}d_{12}^{2}-d_{13}d_{31}\left(d_{22}-\lambda \right)=0.$$

Let $\lambda_{2}=d_{22}$. Then the roots $\lambda_{3}$ and $\lambda_{4}$ of $\left(20\right)$ would be solution of the following equation

$$\left(d_{11}-\lambda \right)\left(d_{33}-\lambda \right)+d_{31}d_{12}^{2}$$

$$=\lambda^{2}-\left(d_{11}+d_{33}\right)\lambda +d_{11}d_{33}+d_{31}d_{12}^{2}=0.$$

The roots of the above equation are

$$\lambda_{3},\;\lambda_{4}=\frac{\left(d_{11}+d_{33}\right)\pm \sqrt{{\left(d_{11}+d_{33}\right)}^{2}-4\left(d_{11}d_{33}+d_{31}d_{12}^{2}\right)}}{2}$$

$$=\frac{\left(d_{11}+d_{33}\right)\pm \sqrt{{\left(d_{11}-d_{33}\right)}^{2}-4d_{31}d_{12}^{2}}}{2},$$

when

$${\left(d_{11}-d_{33}\right)}^{2}\geq 4d_{31}d_{12}^{2}.$$

Moreover, for $d_{22}\geq 0$, $\left(d_{11}+d_{33}\right)\geq 0$ and $d_{11}d_{33}+d_{31}d_{12}^{2}\geq 0$ we get that the matrices $A_{i}$ have different sign of eigenvalues, i.e., in this case $P_{i}$ are saddle points. □

**Theorem 5.**  *Let the Conditions 2 holds. The point $P_{3}$ is a saddle point.*

**Proof.**  The eigenvalues of the matrices $A_{3}$ can found as the solutions of the following equations

(21) $$A_{3}-\lambda I=\left[\begin{array}{cccc} d_{11}-\lambda & d_{12} & d_{13} & 0\\ 0 & d_{22}-\lambda & d_{23} & 0\\ d_{31} & 0 & d_{33}-\lambda & 0\\ 0 & dn & 0 & -c-\lambda \end{array}\right],$$

where $d_{ij}$ are defined by $\left(18\right)$. Hence

$$A_{3}-\lambda I=\left[\begin{array}{cccc} d_{11}-\lambda & 0 & 0 & 0\\ 0 & d_{22}-\lambda & d_{23} & 0\\ 0 & 0 & d_{33}-\lambda & 0\\ 0 & dn & 0 & -c-\lambda \end{array}\right]$$

(22) $$=\left(d_{11}-\lambda \right)\left[\begin{array}{ccc} d_{22}-\lambda & d_{23} & 0\\ 0 & d_{33}-\lambda & 0\\ dn & 0 & -c-\lambda \end{array}\right]$$

$$=-\left(c+\lambda \right)\left(d_{11}-\lambda \right)\left(d_{22}-\lambda \right)\left(d_{33}-\lambda \right)=0.$$

Hence, $\lambda_{0}=-c$, $\lambda_{1}=d_{11}$, $\lambda_{2}=d_{22}$ and $\lambda_{3}=d_{33}$ are eigenvalues of $A_{3}$. Since $d_{11}=\frac{\beta k_{2}r_{2}}{k_{2}r_{2}+k_{1}}+\beta_{1}$ is positive, $d_{22}=r_{2}\left(1-2k_{2}^{-1}k_{2}r_{2}\right)$ is negative when $2k_{2}^{-1}k_{2}r_{2}>1$, and $d_{33}=-d_{2}$ is negative, we obtain that $P_{3}$ is a saddle point.□

## 4. Lyapunov Stability of Equilibria Points

In this section we show the following results:

**Theorem 6.**  *The system $\left(5\right)$ is not stable at the equilibria point $P_{0}\left(0\right)$ in the Lyapunov sense.*

**Proof.**  Indeed, since the one of eigenvalue of the linearized matrix with respect to equilibria point $P_{0}\left(0\right)$ is positive, we get that the system $\left(5\right)$ is not stable at the equilibria point $P_{0}\left(0\right)$. □

Now, we consider the equilibria points $P_{i}$ and prove the following result:

**Theorem 7.**  *Assume that the Conditions 2 and 3 are satisfied. Then the system $\left(5\right)$ is asymptotically stable at the equilibria points $P_{i}$ in the sense of Lyapunov.*

**Proof.**  Let $A_{i}$ be the linearized matrix with respect to equilibria point $P_{i}$ defined by $\left(15\right)$, i.e.,

$$A_{i}=\left[\begin{array}{cccc} d_{11}\left(P_{i}\right) & d_{12}\left(P_{i}\right) & d_{13}\left(P_{i}\right) & 0\\ 0 & d_{22}\left(P_{i}\right) & d_{23}\left(P_{i}\right) & 0\\ d_{31}\left(P_{i}\right) & 0 & d_{33}\left(P_{i}\right) & 0\\ 0 & dn & 0 & -c \end{array}\right],$$

where $d_{kj}=d_{kj}\left(P_{i}\right)$ are defined by $\left(19\right).$

Consider the Lyapunov equation

(23) $$B_{i}A_{i}+A_{i}^{T}B_{i}=-I,$$

where

(24) $$B_{i}=\left[\begin{array}{cccc} b_{11} & b_{12} & b_{13} & b_{14}\\ b_{21} & b_{22} & b_{23} & b_{24}\\ b_{31} & b_{32} & b_{33} & b_{34}\\ b_{41} & b_{42} & b_{43} & b_{44} \end{array}\right],\;b_{kj}=b_{kj}\left(i\right),\;b_{kj}=b_{jk},$$

It is clear that

$$B_{i}A_{i}=\left[\begin{array}{cccc} b_{11} & b_{12} & b_{13} & b_{14}\\ b_{21} & b_{22} & b_{23} & b_{24}\\ b_{31} & b_{32} & b_{33} & b_{34}\\ b_{41} & b_{42} & b_{43} & b_{44} \end{array}\right]\left[\begin{array}{cccc} d_{11} & d_{12} & d_{13} & 0\\ 0 & d_{22} & d_{23} & 0\\ d_{31} & 0 & d_{33} & 0\\ 0 & d_{1}n & 0 & -c \end{array}\right]=\left[c_{kj}\right],$$

$$A_{i}^{T}B_{i}=\left[\begin{array}{cccc} d_{11} & 0 & d_{31} & 0\\ d_{12} & d_{22} & 0 & d_{1}n\\ d_{13} & d_{23} & d_{33} & 0\\ 0 & 0 & 0 & -c \end{array}\right]=\left[\begin{array}{cccc} b_{11} & b_{12} & b_{13} & b_{14}\\ b_{21} & b_{22} & b_{23} & b_{24}\\ b_{31} & b_{32} & b_{33} & b_{34}\\ b_{41} & b_{42} & b_{43} & b_{44} \end{array}\right]=\left[l_{kj}\right],$$

where

$$c_{11}=d_{11}b_{11}+d_{31}b_{13},\;c_{12}=d_{12}b_{11}+d_{22}b_{12}+dnb_{14},$$

$$c_{13}=d_{13}b_{11}+d_{23}b_{12}+d_{33}b_{13},\;c_{14}=-cb_{14},$$

$$c_{21}=d_{11}b_{12}+d_{31}b_{23},\;c_{22}=d_{12}b_{12}+d_{22}b_{22}+dnb_{24},$$

$$c_{23}=d_{13}b_{12}+d_{23}b_{22}+d_{33}b_{23},\;c_{24}=-cb_{24},$$

$$c_{31}=d_{11}b_{13}+d_{31}b_{33},\;c_{32}=d_{12}b_{13}+d_{22}b_{23}+dnb_{34},\;$$

$$c_{33}=d_{13}b_{13}+d_{23}b_{23}+d_{33}b_{33},\;c_{34}=-cb_{34},$$

$$c_{41}=d_{11}b_{14}+d_{31}b_{34},\;c_{42}=d_{12}b_{14}+d_{22}b_{24}+dnb_{44},\;$$

$$c_{43}=d_{13}b_{14}+d_{23}b_{24}+d_{33}b_{34},c_{44}=-cb_{44},\;$$

$$l_{11}=d_{11}b_{11}+d_{31}b_{31},\;l_{12}=d_{11}b_{12}+d_{31}b_{32},\;l_{13}=d_{11}b_{13}+d_{31}b_{33},$$

$$l_{14}=d_{11}b_{14}+d_{31}b_{34},\;l_{21}=d_{12}b_{11}+d_{22}b_{21}+dnb_{14},\;$$

$$l_{22}=d_{12}b_{12}+d_{22}b_{22}+dnb_{42},\;l_{23}=d_{12}b_{13}+d_{22}b_{23}+dnb_{43},\;$$

$$l_{24}=d_{12}b_{14}+d_{22}b_{24}+dnb_{44},\;l_{31}=d_{13}b_{11}+d_{23}b_{21}+d_{33}b_{31},$$

$$l_{32}=d_{13}b_{12}+d_{23}b_{22}+d_{33}b_{32},\;l_{33}=d_{13}b_{13}+d_{23}b_{23}+d_{33}b_{33},$$

$$l_{34}=d_{13}b_{14}+d_{23}b_{24}+d_{33}b_{34},\;l_{41}=-cb_{41},\;l_{42}=-cb_{42},$$

$$\;l_{43}=-cb_{43},\;l_{44}=-cb_{44}.$$

Since $b_{kj}=b_{jk}$ the matrix equation $\left(24\right)$ reduced to the following system of equations with respect to $b_{kj}$

$$c_{kj}+d_{kj}=\left\{\begin{array}{c} -1\;\mathrm{for}\;k=j\\ 0\;\mathrm{for}\;k\neq j \end{array}\right\}.$$

i.e., we obtain the system of algebraic equations with respect to $b_{11}$, $b_{12}$, $b_{13}$, $b_{14}$, $b_{22}$, $b_{23}$, $b_{24}$, $b_{33}$ and $b_{44};$

$$d_{11}b_{11}+d_{31}b_{13}=-\frac{1}{2},\;d_{12}b_{11}+\left(d_{11}+d_{22}\right)b_{12}+dnb_{14}+d_{31}b_{23}=0,\;$$

$$d_{13}b_{11}+d_{23}b_{12}+\left(d_{33}+d_{11}\right)b_{13}+d_{31}b_{33}=0,\;\left(d_{11}-c\right)b_{14}+d_{31}b_{34}=0,$$

$$d_{12}b_{12}+d_{22}b_{22}+d_{1}nb_{24}=-\frac{1}{2},\;d_{13}b_{12}+d_{23}b_{22}+\left(d_{22}+d_{33}\right)b_{23}+d_{12}b_{13}+dnb_{34}=0,$$

$$\left(d_{22}-c\right)b_{24}+d_{12}b_{14}+dnb_{44}=0,\;cb_{44}=\frac{1}{2}.$$

$$d_{13}b_{13}+d_{23}b_{23}+d_{33}b_{33}=-\frac{1}{2},\;\left(d_{33}-c\right)b_{34}+d_{13}b_{14}+d_{23}b_{24}=0.$$

We obtain the following matrix equation

(25) $$GB=-\frac{1}{2}J,\;G=G\left(i\right),$$

where

$$G=\left[\begin{array}{cccccccccc} d_{11} & 0 & d_{31} & 0 & 0 & 0 & 0 & 0 & 0 & 0\\ d_{12} & d_{11}+d_{22} & 0 & d_{1}n & 0 & d_{31} & 0 & 0 & 0 & 0\\ d_{13} & d_{23} & d_{11}+d_{33} & 0 & 0 & d_{31} & 0 & 0 & 0 & 0\\ 0 & 0 & 0 & d_{11}-c & 0 & 0 & 0 & d_{31} & 0 & 0\\ 0 & d_{12} & 0 & 0 & d_{22} & 0 & d_{1}n & 0 & 0 & 0\\ 0 & d_{13} & d_{12} & 0 & d_{23} & d_{22}+d_{33} & 0 & 0 & d_{1}n & 0\\ 0 & 0 & 0 & d_{12} & 0 & d_{22}-c & 0 & 0 & 0 & dn\\ 0 & 0 & d_{13} & 0 & 0 & d_{23} & 0 & d_{33} & 0 & 0\\ 0 & 0 & 0 & d_{13} & 0 & 0 & d_{23} & 0 & a_{33}-c & 0\\ 0 & 0 & 0 & 0 & 0 & 0 & 0 & 0 & 0 & c \end{array}\right],$$

$$B=\left[\begin{array}{c} b_{11}\\ b_{12}\\ b_{13}\\ b_{14}\\ b_{22}\\ b_{23}\\ b_{24}\\ b_{33}\\ b_{34}\\ b_{44} \end{array}\right],\;-\frac{1}{2}J_{10}=\left[\begin{array}{c} -\frac{1}{2}\\ 0\\ 0\\ 0\\ -\frac{1}{2}\\ 0\\ 0\\ \frac{1}{2}\\ 0\\ \frac{1}{2} \end{array}\right].$$

Let Det G≠0. Then the system $\left(24\right)$ have a solution

$$b_{11}=\frac{DetG_{1}}{DetG},\;b_{12}=b_{21}=\frac{DetG_{2}}{DetG},\;b_{13}=b_{31}=\frac{DetG_{3}}{DetG},$$

$$...,\;b_{34}=b_{43}=\frac{DetG_{9}}{DetG},\;b_{44}=\frac{DetG_{10}}{DetG},$$

where $G_{k}$ are the additional matrices obtained from the main matrix *G* by replacing *k*-th column with $-\frac{1}{2}J_{10}$. We assume that $a_{kj}$, *c*, λ such that

(26) $$b_{kk}>0,\;k=1,2,3,4.$$

Consider the quadratic function

$$V_{i}\left(x\right)=X^{T}B_{i}X=b_{11}x_{1}^{2}+b_{22}x_{2}^{2}+2b_{12}x_{1}x_{2}+2b_{13}x_{1}x_{3}+$$

$$b_{33}x_{3}^{2}+2b_{23}x_{2}x_{3}+2b_{24}x_{2}x_{4}+b_{44}x_{4}^{2}=$$

(27) $$=\frac{1}{2}b_{11}{\left(x_{1}+2\frac{b_{12}}{b_{11}}x_{2}\right)}^{2}+\left(b_{22}-\frac{2b_{12}^{2}}{b_{11}}\right)x_{2}^{2}+$$

$$b_{33}x_{3}^{2}+2b_{23}x_{2}x_{3}+2b_{24}x_{2}x_{4}+b_{44}x_{4}^{2}=$$

$$\frac{1}{2}b_{11}{\left(x_{1}+2\frac{b_{13}}{b_{11}}x_{2}\right)}^{2}+\left(b_{33}-\frac{2b_{13}^{2}}{b_{11}}\right)x_{3}^{2}+$$

$$\frac{1}{2}b_{22}{\left(x_{2}+2\frac{b_{23}}{b_{22}}x_{3}\right)}^{2}+\left(b_{33}-\frac{2b_{23}^{2}}{b_{22}}\right)x_{3}^{2}+$$

$$\frac{1}{2}b_{22}{\left(x_{2}+2\frac{b_{23}}{b_{22}}x_{4}\right)}^{2}+\left(b_{44}-\frac{2b_{24}^{2}}{b_{22}}\right)x_{4}^{2}.$$

From $\left(25\right)$ we see that $V_{i}\left(x\right)\geq 0$, when the following hold

(28) $$b_{22}\geq \frac{2b_{12}^{2}}{b_{11}},\;b_{33}\geq \frac{2b_{13}^{2}}{b_{11}},\;b_{33}\geq \frac{2b_{23}^{2}}{b_{22}},\;b_{44}\geq \frac{2b_{24}^{2}}{b_{22}}.$$

Thus, $V_{i}\left(x\right)$ are positive defined Lyapunov functions. By ([12] Corollary 8.2) we need now to determine the domains $\Omega_{i}$ on which ${\dot{V}}_{i}\left(x\right)$ is negatively defined. By assuming $x_{k}\geq 0$, k=1,2,3,4 we will find the solution set of the following inequality

(29) $${\dot{V}}_{i}\left(x\right)=\sum_{j=1}^{4}\frac{\partial V_{i}}{\partial x_{j}}f_{j}\left(x\right)=$$

$$=2B_{1}\left(x\right)\left[\frac{\beta x_{2}}{x_{2}+k_{1}}+\beta_{1}x_{4}-q_{13}x_{3}\right]x_{1}+$$

$$2B_{2}\left(x\right)\left[r_{2}\left(1-2k_{2}^{-1}x_{2}\right)+\beta_{2}x_{4}-q_{23}x_{3}\right]x_{2}+$$

$$2B_{3}\left(x\right)\left[\frac{d_{3}x_{1}}{x_{1}+k_{3}}-q_{32}x_{1}-d_{3}\right]x_{3}+2B_{3}\left(x\right)\left(dnx_{2}-cx_{4}\right)\leq 0,$$

where

$$B_{j}\left(x\right)=\sum_{k=1}^{4}b_{jk}x_{k},\;j=1,2,3,4.$$

It is clear to see that $\left(29\right)$ holds, when

$$B_{1}\left(x\right)\geq 0,\;\frac{\beta x_{2}}{x_{2}+k_{1}}+\beta_{1}x_{4}-q_{13}x_{3}\leq 0,$$

$$B_{2}\left(x\right)\geq 0,\;r_{2}\left(1-2k_{2}^{-1}x_{2}\right)+\beta_{2}x_{4}-q_{23}x_{3}\leq 0,$$

$$B_{3}\left(x\right)\geq 0,\;\frac{d_{3}x_{1}}{x_{1}+k_{3}}-q_{32}x_{1}\leq d_{3},$$

$$B_{4}\left(x\right)\geq 0,\;dnx_{2}-cx_{4}\leq 0$$

or

$$B_{1}\left(x\right)\leq 0,\;\frac{\beta x_{2}}{x_{2}+k_{1}}+\beta_{1}x_{4}-q_{13}x_{3}\geq 0,$$

$$B_{2}\left(x\right)\leq 0,\;r_{2}\left(1-2k_{2}^{-1}x_{2}\right)+\beta_{2}x_{4}-q_{23}x_{3}\geq 0,$$

$$B_{3}\left(x\right)\leq 0,\;\frac{d_{3}x_{1}}{x_{1}+k_{3}}-q_{32}x_{1}\geq d_{3},$$

$$B_{4}\left(x\right)\leq 0,\;dnx_{2}-cx_{4}\geq 0,$$

i.e., ${\dot{V}}_{i}\left(x\right)\leq 0$ in the following domains

$$\Omega_{i1}=\left\{x\in R_{+}^{4},\right.B_{1}\left(x\right)\geq 0,\;\frac{\beta x_{2}}{x_{2}+k_{1}}+\beta_{1}x_{4}-q_{13}x_{3}\leq 0,$$

$$B_{2}\left(x\right)\geq 0,\;r_{2}\left(1-2k_{2}^{-1}x_{2}\right)+\beta_{2}x_{4}-q_{23}x_{3}\leq 0,\;B_{3}\left(x\right)\geq 0,\;$$

(30) $$\left[\frac{d_{3}}{x_{1}+k}-q_{32}\right]x_{1}\leq d_{3},\;B_{4}\left(x\right)\geq 0,\;x_{2}\leq \left.\frac{c}{dn}x_{4}\right\},$$

$$\Omega_{i1}=\left\{x\in R_{+}^{4},\right.B_{1}\left(x\right)\leq 0,\;\frac{\beta x_{2}}{x_{2}+k_{1}}+\beta_{1}x_{4}-q_{13}x_{3}\geq 0,$$

$$B_{2}\left(x\right)\leq 0,\;r_{2}\left(1-2k_{2}^{-1}x_{2}\right)+\beta_{2}x_{4}-q_{23}x_{3}\geq 0,$$

$$B_{3}\left(x\right)\leq 0,\;\left[\frac{d_{2}}{x_{1}+k}-q_{33}\right]x_{1}\geq d_{1},\;B_{4}\left(x\right)\geq 0,\left.x_{2}\geq \frac{c}{dn}x_{4}\right\}.$$

That is the system $\left(5\right)$ is asymptotically stable at the equilibria points $E_{i}$ on the domains

(31) $$\Omega_{i}=\Omega_{i1}\cup \Omega_{i2}.$$

□

**Figure 1:**
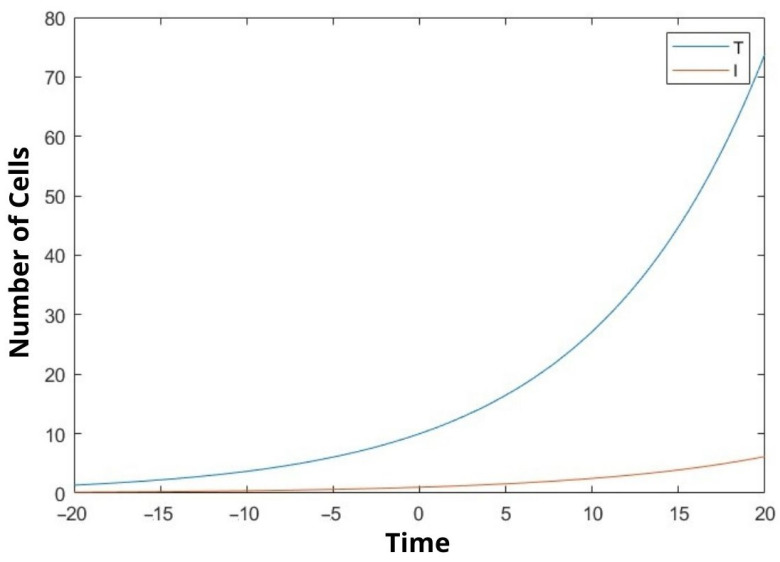
We compare the cancer cells (T(t)) and the infected cells (I(t)).

**Figure 2:**
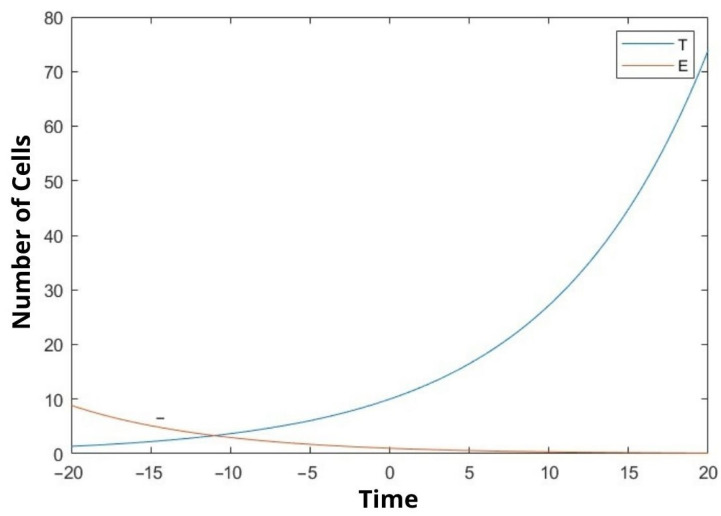
We compare the cancer cells (T(t)) and the effector immune cells (E(t)).

**Figure 3:**
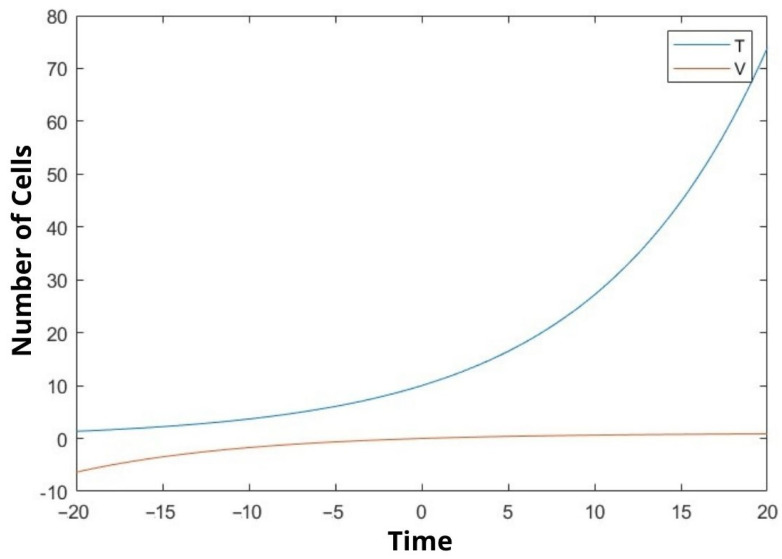
We compare the cancer cells (T(t)) and the free viruses cells (V(t)).

In our study, we mathematically demonstrated the relationship between uninfected cells, infected cells, effector immune cells, and free viruses with a dynamic model. We examined the stability analysis of the system and the Lyapunov stability of the equilibrium points. Clinical studies have not yet been conducted. We tried to make a mathematical determination. In Figure 1, Figure 2 and Figure 3, We compare the cancer cells with the infected cells and the effector immune cells. When the cancer cells increase rapidly, the infected cells and the free viruses cells do not increase so quickly in Figure 1 and Figure 3. On the other hand, when the cancer cells increase rapidly, the effector immune cells decrease rapidly in Figure 2. The constants in the equations are taken as 0.1 The moment of time taken after the beginning of time, that is time zero, is called positive time, while the time taken before the beginning of time is negative. There is a negative time from ten on the chart. Because this is a function, it has a corresponding value in the negative values of the x coordinate. The time in the graphs is taken as unit time.

## 5. Basin of Attractions

In this section, we will derive the domain attraction sets of the problem $\left(3\right)$ and $\left(4\right)$ at attractor points $E_{i}$. Lyapunov’s method can be used to find the region of attraction or an estimate of it. We show in this section the following results:

**Theorem 8.**  *Assume that the Condition 2 is satisfied. Then the basin of multiphase attraction set of $\left(3\right)$–$\left(4\right)$ at $x\left(i\right)=P_{i}$ belongs to the sets $\Omega_{C}\left(i\right)\subset \Omega_{i}$ and*

$$\Omega_{C}\left(i\right)=\left\{x\in R_{+}^{4}:\;V_{i}\left(x\right)\leq C_{i}\;\right\},$$

*here positive constants $C_{i}$ are defined in bellow, $\Omega_{i}$ were defined by $\left(31\right)$.*

**Proof.**  We are interested in the largest sets $\Omega_{C}\left(i\right)\subset \Omega_{i}$ that we can determine the largest value for the constants $C_{i}$ such that $\Omega_{C_{i}}\left(i\right)\subset D\left(V_{i}\right)$, where

$$D\left(V_{i}\right)=\left\{x\in R^{4},\;V_{i}\left(x\right)\geq 0,\;{\dot{V}}_{i}\left(x\right)<0\right\}.$$

Let us now, find the sets $\Omega_{C_{i}}\left(i\right)\subset B_{r_{i}}\left(x\left(i\right)\right)$, where

$$C_{i}<\min_{\left|x-x\left(i\right)\right|=r_{i}}V_{i}\left(x\right)=\lambda_{\min}\left(A_{i}\right)r_{i}^{2},$$

here $A_{i}$ were defined by $\left(21\right)$, $\lambda_{\min}\left(A_{i}\right)$ denote the minimum eigenvalues of the corresponding matrices $A_{i}$. Moreover, for some $C_{i}>0$ the inclusion $\Omega_{C_{i}}\left(i\right)\subset \Omega_{i}$ means the existence of $C_{i}>0$ such that $x\in \Omega_{C_{i}}\left(i\right)$ implies $x\in G_{i1}\cup G_{i2}$. Here $G_{i1}$, $G_{i2}$ are defined by

(32) $$\;G_{i1}=\left\{x\in R_{+}^{4},\;B_{k}\left(x\right)\geq 0,\right.k=1,2,3,4,$$

$$x_{3}\geq \frac{1}{q_{13}}\left[\frac{\beta x_{2}}{x_{2}+k_{1}}+\beta_{1}x_{4}\right],\;x_{4}+r_{2}\leq \frac{1}{\beta_{2}}\left[2r_{2}k_{2}^{-1}x_{2}+q_{23}x_{3}\right],$$

$$\left.x_{1}\leq \frac{k_{3}d_{2}}{d_{1}-k_{3}q_{33}},\;x_{2}\leq \frac{c}{dn}x_{4}\right\},\;d_{1}>k_{3}q_{33},$$

(33) $$\;G_{i2}=\left\{x\in R_{+}^{4},\;B_{k}\left(x\right)\leq 0,\right.k=1,2,3,4,$$

$$x_{3}\leq \frac{1}{q_{13}}\left[\frac{\beta x_{2}}{x_{2}+k_{1}}+\beta_{1}x_{4}\right],\;x_{4}+r_{2}\geq \frac{1}{\beta_{2}}\left[2r_{2}k_{2}^{-1}x_{2}+q_{23}x_{3}\right],$$

$$\left.x_{1}\geq \frac{k_{3}d_{2}}{d_{1}-k_{3}q_{33}},\;x_{2}\geq \frac{c}{dn}x_{4}\right\}.$$

From $\left(32\right)$ we deduced that

(34) $$\;D_{i1}=\left\{x\in R_{+}^{4},\;B_{k}\left(x\right)\geq 0,\right.k=1,2,3,4,$$

$$x_{4}\leq \frac{q_{13}}{\mu_{1}}x_{3}-x_{2},\;x_{4}\leq \frac{\mu_{2}}{\beta_{2}}\left(x_{2}+x_{3}\right),\;x_{2}\leq \frac{q_{13}}{\mu_{1}}x_{3},$$

$$\left.x_{1}\leq \frac{k_{3}d_{2}}{d_{1}-k_{3}q_{33}},\;x_{2}\leq \frac{c}{dn}x_{4}\right\}\subset \;G_{i1},\;d_{1}k_{3}q_{33}$$

(35) $$\;{\bar{D}}_{i1}=\left\{x\in R_{+}^{4},\;B_{k}\left(x\right)\geq 0,\right.k=1,2,3,4,$$

$$\left(1+\frac{c}{dn}\right)x_{4}\leq \frac{q_{13}}{\mu_{1}}x_{3},\;x_{2}\leq \frac{q_{13}}{\mu_{1}}x_{3},\left.x_{1}\leq \frac{k_{3}d_{2}}{d_{1}-k_{3}q_{33}}\right\}\subset G_{i1},$$

where

$$\mu_{1}=\max\left\{\frac{\beta }{k_{1}},\;\beta_{1}\right\},\;\mu_{2}=\min\left\{2r_{2}k_{2}^{-1},\;q_{23}\right\},\;\frac{\mu_{2}}{\beta_{2}}-\frac{q_{13}}{\mu_{1}}>0.$$

From $\left(34\right)$ and $\left(35\right)$ we have

$$\sum_{k=1}^{4}{\left[x_{k}-x_{k}\left(i\right)\right]}^{2}=\sum_{k=1}^{4}x_{k}^{2}-2x_{k}x_{k}\left(i\right)+{\left(x_{k}\left(i\right)\right)}^{2}\leq r_{i1}^{2},$$

where

$$r_{i1}=\sqrt{2}\left\{{\left[\frac{k_{3}d_{3}}{d_{3}-k_{3}q_{32}}\right]}^{2}+\left[1+{\left(\frac{q_{13}}{\mu_{1}}\right)}^{2}\right]\frac{\beta^{2}}{q_{13}^{2}}\right.+$$

$$\mu_{3}^{2}\frac{\beta^{2}}{q_{13}^{2}}+{\left.{\left(\frac{q_{13}}{\mu_{1}}\right)}^{2}\frac{\beta^{2}}{q_{13}^{2}}\right\}}^{\frac{1}{2}}+{\left(x_{k}\left(i\right)\right)}^{2}.$$

Hence,

$$\;{\tilde{\Omega }}_{i1}=\left\{\left\{x\in R_{+}^{4},\right.B_{k}\left(x\right)\geq 0,\sum_{k=1}^{4}{\left[x_{k}-x_{k}\left(i\right)\right]}^{2}\leq r_{i1}^{2}\right\}\subset \Omega_{i1}.\;$$

Then we obtain

$$C_{i1}<\min_{\left|x\right|=r_{i1}}V_{i}\left(x\right).$$

Moreover, consider now, the case with domain $G_{i2}$ defined by $\left(33\right)$. It is clear to see that

(36) $$\;D_{i2}=\left\{x\in R_{+}^{4},\;B_{k}\left(x\right)\leq 0,\right.k=1,2,3,4,$$

$$x_{1}=\frac{k_{3}d_{2}}{d_{1}-k_{3}q_{33}},\;x_{2}=\frac{c}{dn}x_{4},\;x_{3}\leq \frac{\beta_{1}}{q_{13}}x_{4},$$

$$\;\beta_{2}x_{4}\geq \left[2r_{2}k_{2}^{-1}\frac{c}{dn}x_{4}+q_{23}x_{3}\right],\left.d_{1}>k_{3}q_{33}\right\}.$$

Let we put

$$\left[\beta_{2}-2r_{2}k_{2}^{-1}\frac{c}{dn}\right]x_{4}=q_{23}x_{3}.$$

From $\left(34\right)$ and $\left(36\right)$ then we have

$$\sum_{k=1}^{4}{\left[x_{k}-x_{k}\left(i\right)\right]}^{2}=\sum_{k=1}^{4}x_{k}^{2}-2x_{k}x_{k}\left(i\right)+{\left(x_{k}\left(i\right)\right)}^{2}$$

$$\leq 2\sum_{k=1}^{4}x_{k}^{2}+{\left(x_{k}\left(i\right)\right)}^{2}\leq 2\left\{{\left(\frac{k_{3}d_{2}}{d_{1}-k_{3}q_{33}}\right)}^{2}+\left[{\left(\frac{c}{dn}\right)}^{2}\right.\right.+$$

$$\left.\eta_{1}^{2}+1\right]x_{4}^{2}\leq r_{i2}^{2},$$

where

$$\eta_{1}=\min\left\{\frac{\beta_{1}}{q_{13}},\;\left[\beta_{2}-2r_{2}k_{2}^{-1}\frac{c}{dn}\right]\frac{1}{q_{23}}\right\},$$

here we assume

$$\beta_{2}\geq 2r_{2}k_{2}^{-1}\frac{c}{dn}.$$

So,

$$\;{\tilde{\Omega }}_{i1}=\left\{\left\{x\in R_{+}^{4},\right.B_{k}\left(x\right)\leq 0,\sum_{k=1}^{4}{\left[x_{k}-x_{k}\left(i\right)\right]}^{2}\leq r_{i2}^{2}\right\}\subset \Omega_{i2}.\;$$

Then we obtain

$$C_{i2}<\min_{\left|x\right|=r_{i2}}V_{i}\left(x\right).$$

□

## 6. Discussion

Observing the outcome of our research we can say that the results are quite significant. After inspecting the figures (Figure 1, Figure 2 and Figure 3), it is possible to say that rate of increase in cancer cells is proportional to the increase in virus cells while it is inversely proportional to immune cells. Thus, it is possible to say that our model is accurate. It is not natural to expect all the coefficients of the variables taken in the experiment to be 0.1 but we believed the outcome would be more fitting by doing so. Since the equation we are trying to solve here is nonlinear, its exact solution cannot be found. Almost all nonlinear equations lack an exact solution. Hence, we are in the process of finding an approximate solution based on assumptions. Although we have achieved this result by the aforementioned method, our solution is admissible since it supports the foreseen outcome. On the other hand, it is possible to say that further improvements can be made to our model. In the comparison of the solved dynamic system with the literature, it is understood that the results are as expected. The next step will be to try to solve the problem we have solved mathematically with clinical data. Future studies will be aimed at including real data from laboratory settings. This study can be improved by increasing the variable number and adding other appropriate parameters from physiology.

## 7. Conclusions

In this study, the interactions between cancer cells, viruses, infected cells, and effector immune cells were discussed. In particular, we graphically showed the relationship between cancer cells and the other three cells at certain values. Equilibrium points were found depending on the constants. Stability analyzes of equilibrium points were examined. In addition, Lyapunov stability analysis of the equilibrium points was also performed. We hope that the established mathematical model will be useful to decision-makers in the field of healthcare. We revealed the comparison between cancer cells, the infected cells and the effector immune cells. When the cancer cells increase rapidly, the infected cells and the free virus cells do not increase so quickly (see Figure 1 and Figure 3). On the other hand, when the cancer cells increase rapidly, the effector immune in Figure 2 is significant. To the best of the author’s knowledge, this topic is shown for the first time. The model was developed to assist protocols applied in the treatment of cancer patients. It is aimed at choosing the factors affecting the coefficient of the equations in the most appropriate way and to help the patient receive better treatment.

## Data Availability

The simulation data presented in this study are available on request from the corresponding author.
